# Supplementary material for: The yeast genome is globally accessible in living cells
Source: Nat Struct Mol Biol. 2024 Nov 25;32(2):247–56. doi: 10.1038/s41594-024-01318-2 (PMC11832417; doi:10.1038/s41594-024-01318-2)
Supplement: Supplementary file 1 — Reporting Summary [file 41594_2024_1318_MOESM1_ESM.pdf]

nature portfolio

Corresponding author(s): David J. Clark

Last updated by author(s): Mar 28, 2024

Reporting Summary

Nature Portfolio wishes to improve the reproducibility of the work that we publish. This form provides structure for consistency and transparency in reporting. For further information on Nature Portfolio policies, see our Editorial Policies and the Editorial Policy Checklist.

Statistics

For all statistical analyses, confirm that the following items are present in the figure legend, table legend, main text, or Methods section.

n/a	Confirmed	
☒	☐	The exact sample size (*n*) for each experimental group/condition, given as a discrete number and unit of measurement
☐	☒	A statement on whether measurements were taken from distinct samples or whether the same sample was measured repeatedly
☒	☐	The statistical test(s) used AND whether they are one- or two-sided<br>*Only common tests should be described solely by name; describe more complex techniques in the Methods section.*
☒	☐	A description of all covariates tested
☒	☐	A description of any assumptions or corrections, such as tests of normality and adjustment for multiple comparisons
☐	☒	A full description of the statistical parameters including central tendency (e.g. means) or other basic estimates (e.g. regression coefficient) AND variation (e.g. standard deviation) or associated estimates of uncertainty (e.g. confidence intervals)
☒	☐	For null hypothesis testing, the test statistic (e.g. *F*, *t*, *r*) with confidence intervals, effect sizes, degrees of freedom and *P* value noted<br>*Give P values as exact values whenever suitable.*
☒	☐	For Bayesian analysis, information on the choice of priors and Markov chain Monte Carlo settings
☒	☐	For hierarchical and complex designs, identification of the appropriate level for tests and full reporting of outcomes
☐	☒	Estimates of effect sizes (e.g. Cohen's *d*, Pearson's *r*), indicating how they were calculated

Our web collection on statistics for biologists contains articles on many of the points above.

Software and code

Policy information about availability of computer code

Data collection	Illumina data were aligned to the sacCer3 version of the yeast genome (available at the Saccharomyces Genome Database) using Bowtie 2. Nanopore data were processed using Nanopolish v.0.14.0, mapped to sacCer3 using Minimap2 v.2.24, and and sorted and indexed with samtools v.1.17.
Data analysis	Specific Matlab scripts are publicly available at GitHub: https://github.com/clarkda24/Prajapati_et_al_2023.git

For manuscripts utilizing custom algorithms or software that are central to the research but not yet described in published literature, software must be made available to editors and reviewers. We strongly encourage code deposition in a community repository (e.g. GitHub). See the Nature Portfolio guidelines for submitting code & software for further information.

Data

Policy information about availability of data

All manuscripts must include a data availability statement. This statement should provide the following information, where applicable:

- Accession codes, unique identifiers, or web links for publicly available datasets
- A description of any restrictions on data availability
- For clinical datasets or third party data, please ensure that the statement adheres to our policy

All Illumina paired-end and Nanopore sequence data have been deposited in the GEO database with accession number GSE230309 and are publicly available. https://www.ncbi.nlm.nih.gov/geo/query/acc.cgi?acc=GSE230309

Research involving human participants, their data, or biological material

Policy information about studies with human participants or human data. See also policy information about sex, gender (identity/presentation), and sexual orientation and race, ethnicity and racism.

Reporting on sex and gender	No human participants or human data.
Reporting on race, ethnicity, or other socially relevant groupings	No human participants or human data.
Population characteristics	No human participants or human data.
Recruitment	No human participants or human data.
Ethics oversight	No human participants or human data.

Note that full information on the approval of the study protocol must also be provided in the manuscript.

Field-specific reporting

Please select the one below that is the best fit for your research. If you are not sure, read the appropriate sections before making your selection.

☒ Life sciences ☐ Behavioural & social sciences ☐ Ecological, evolutionary & environmental sciences

For a reference copy of the document with all sections, see nature.com/documents/nr-reporting-summary-flat.pdf

Life sciences study design

All studies must disclose on these points even when the disclosure is negative.

Sample size	Two independent biological replicate experiments were performed for each genomic sample.
Data exclusions	Data for GATC sites with neighbouring GATC sites closer than 200 bp on both sides were excluded due to differential loss of short DNA fragments during library preparation. Described in detail in Methods.
Replication	Two independent biological replicate experiments were performed for each genomic sample. Parallel analysis of replicate experiments is provided in Extended Data. All replicate experiments were successful.
Randomization	Randomisation is not applicable to yeast genomics experiments.
Blinding	Blinding is not applicable to yeast genomics experiments

Reporting for specific materials, systems and methods

We require information from authors about some types of materials, experimental systems and methods used in many studies. Here, indicate whether each material, system or method listed is relevant to your study. If you are not sure if a list item applies to your research, read the appropriate section before selecting a response.

Materials & experimental systems

n/a	Involved in the study	
☐	☒	Antibodies
☒	☐	Eukaryotic cell lines
☒	☐	Palaeontology and archaeology
☒	☐	Animals and other organisms
☒	☐	Clinical data
☒	☐	Dual use research of concern
☒	☐	Plants

Methods

n/a	Involved in the study	
☒	☐	ChIP-seq
☒	☐	Flow cytometry
☒	☐	MRI-based neuroimaging

Antibodies

Antibodies used	HRP-conjugated anti-HA antibody 3F10 (Roche 12013819001) used at 1:5,000 dilution in 5% skim milk in TBS (20mM Tris-HCl pH 8.0, 0.5 M NaCl) with 0.1% Tween-20 overnight at 4oC. HRP-conjugated anti-tubulin antibody (Abcam ab-185067; lot GR3407868-1)

diluted 1:20,000 in PBS/0.1% Tween-20 with 0.5% skim milk for 1 h at room temperature with rotation.

Validation

The anti-HA antibody is raised against a haemagglutinin (HA) epitope used for protein tagging. It has been validated by the manufacturer (https://www.sigmaaldrich.com/US/en/product/roche/12013819001#product-documentation). We have observed that this antibody does not give a signal with a yeast strain lacking an HA-tagged protein (as expected). The anti-tubulin antibody was used as a loading control for Western blots. This antibody was validated by the manufacturer for mammalian tubulins (https://www.abcam.com/products/primary-antibodies/hrp-alpha-tubulin-antibody-epr13478b-loading-control-ab185067.html); yeast tubulin is highly conserved and we observed a single band of the expected size (50 kDa).
