# Supplementary figures and images for: The yeast genome is globally accessible in living cells
Source: Nat Struct Mol Biol. 2024 Nov 25;32(2):247–56. doi: 10.1038/s41594-024-01318-2 (PMC11832417; doi:10.1038/s41594-024-01318-2)

Fig. 1a

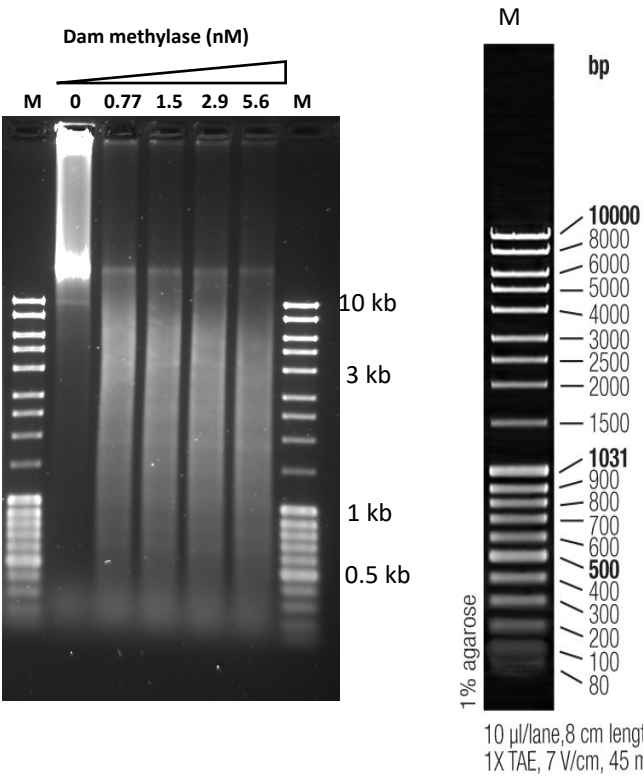

Supplement: Supplementary file 4 — Unprocessed gel. [file 41594_2024_1318_MOESM4_ESM.pdf]

**Fig. 2b**

**Anti-HA blot**

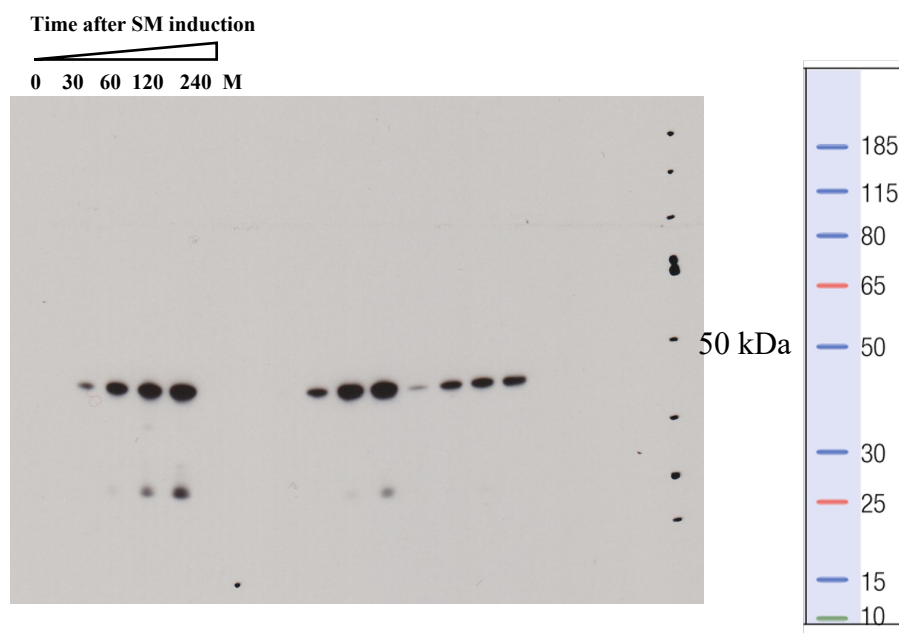

**Anti-tubulin blot**

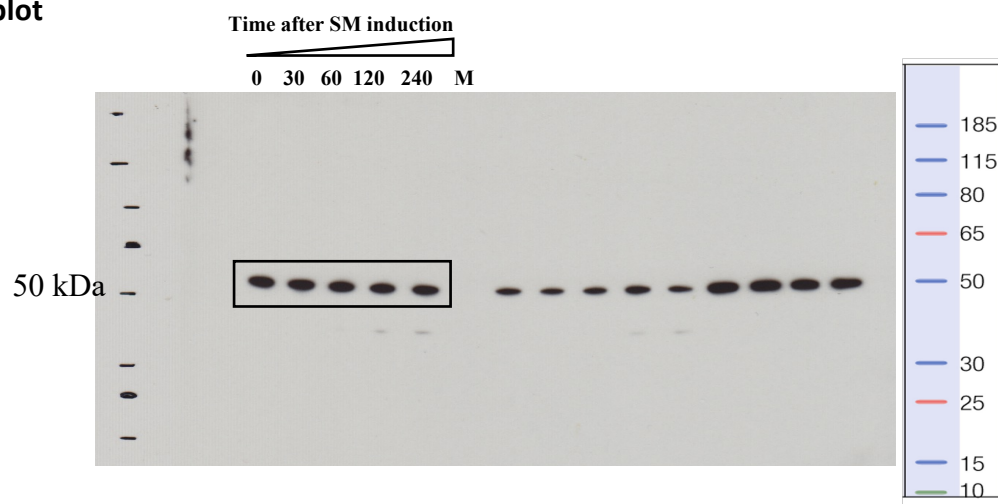

Fig. 2c

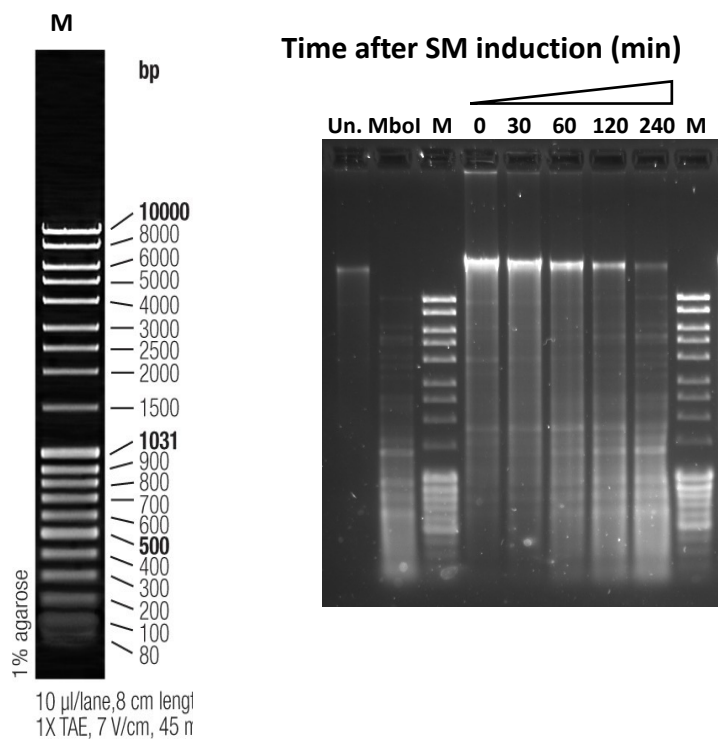

Supplement: Supplementary file 6 — Unprocessed western blots and gel. [file 41594_2024_1318_MOESM6_ESM.pdf]

Fig. 3b

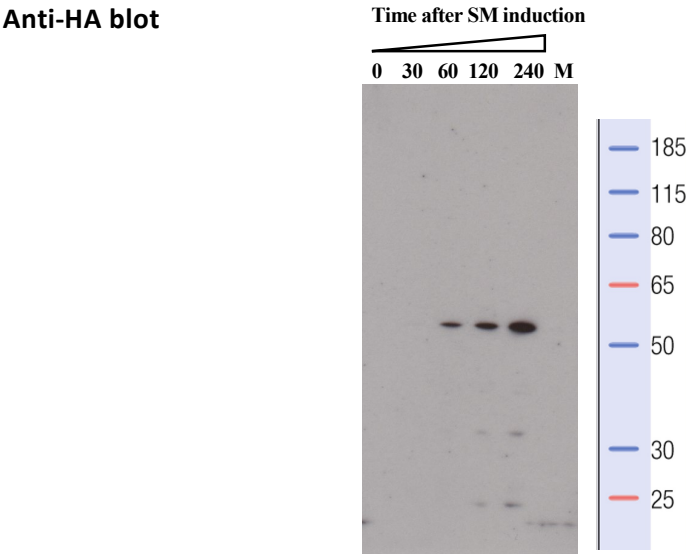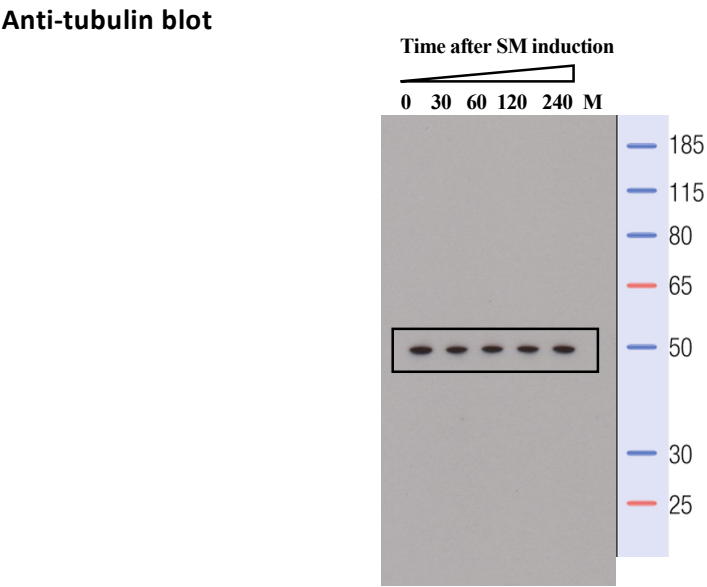

Supplement: Supplementary file 8 — Unprocessed western blots. [file 41594_2024_1318_MOESM8_ESM.pdf]

Fig. 4a

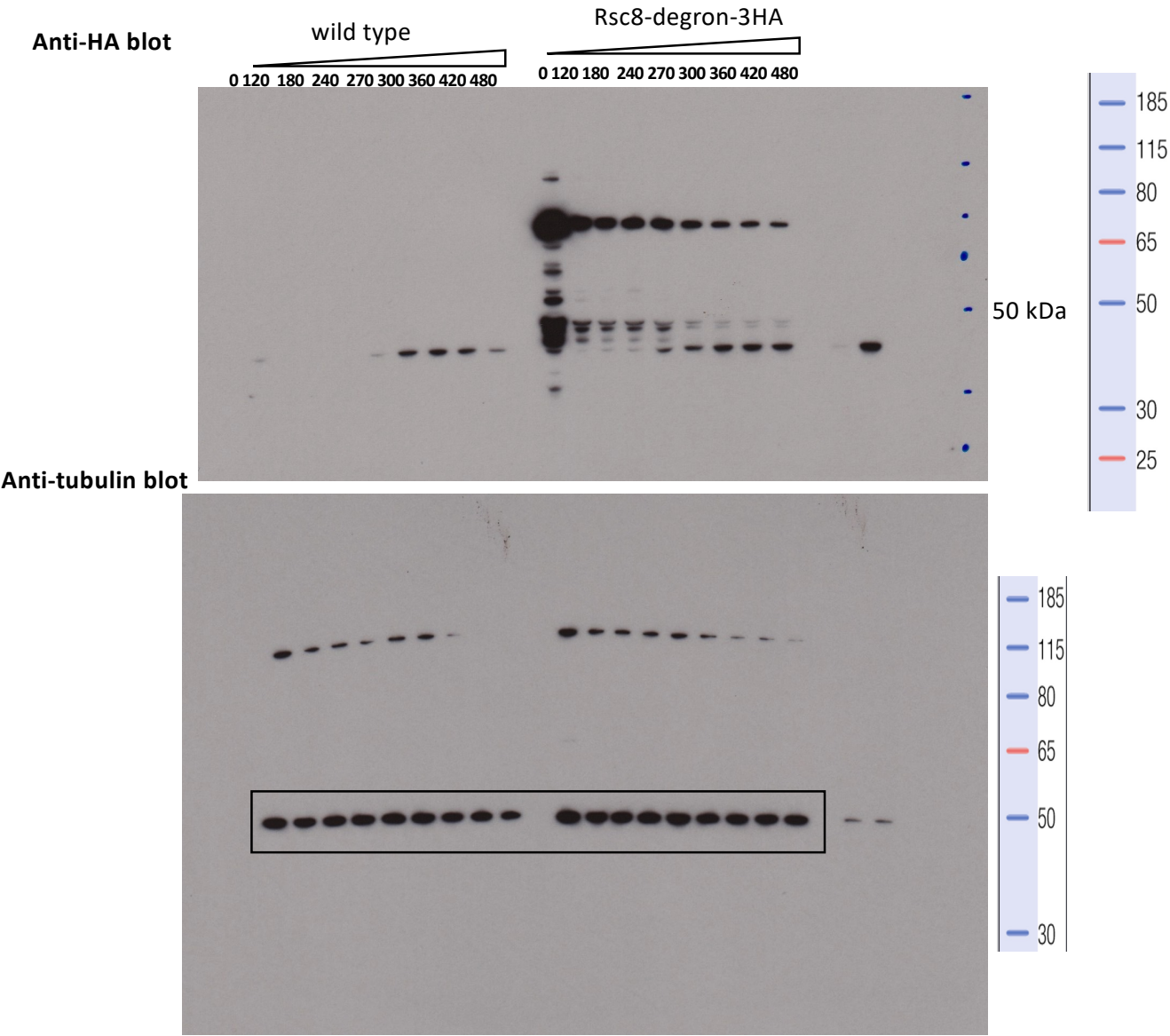

Fig. 4b

Anti-HA blot

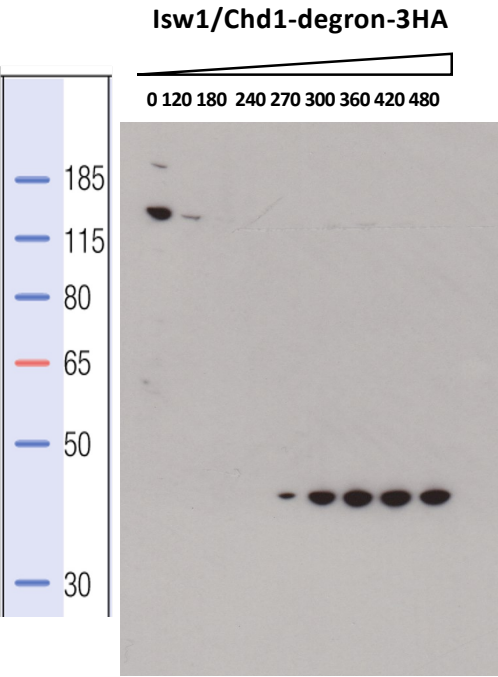

Anti-tubulin blot

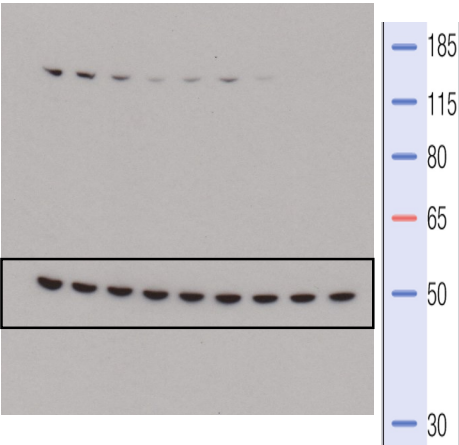

Supplement: Supplementary file 10 — Unprocessed western blots. [file 41594_2024_1318_MOESM10_ESM.pdf]

Extended Data Fig. 3a

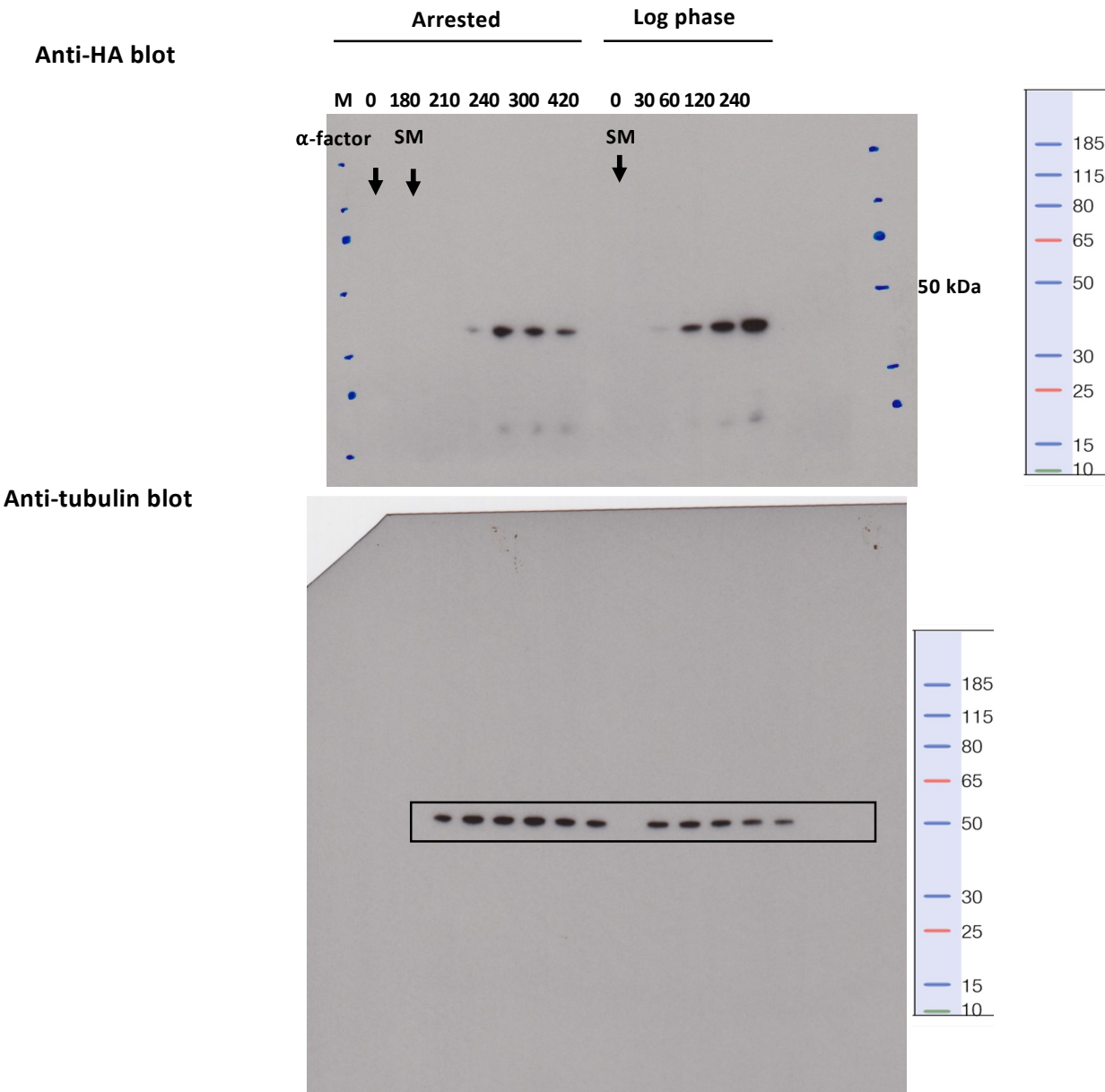

Extended Data Fig. 3b

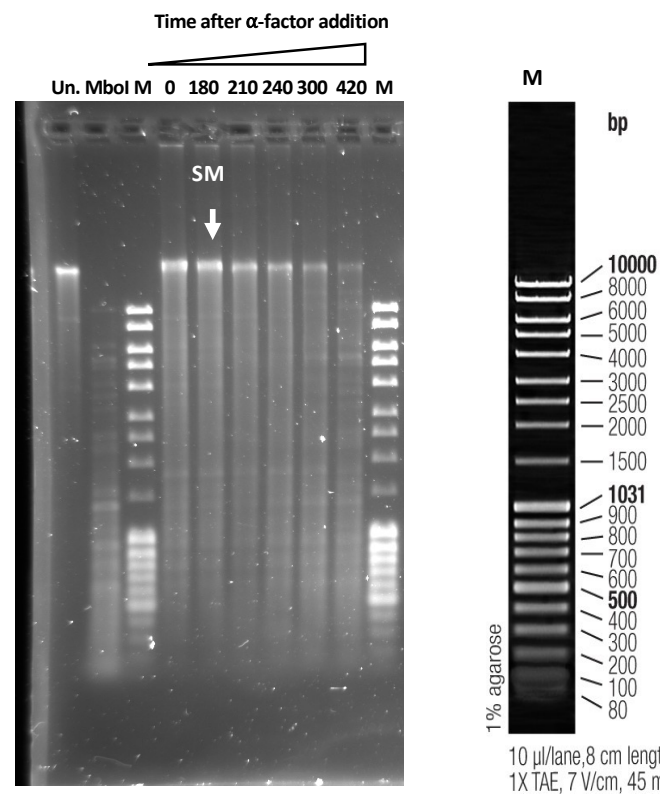

Supplement: Supplementary file 14 — Unprocessed western blots and gel. [file 41594_2024_1318_MOESM14_ESM.pdf]

Extended Data Fig. 4c

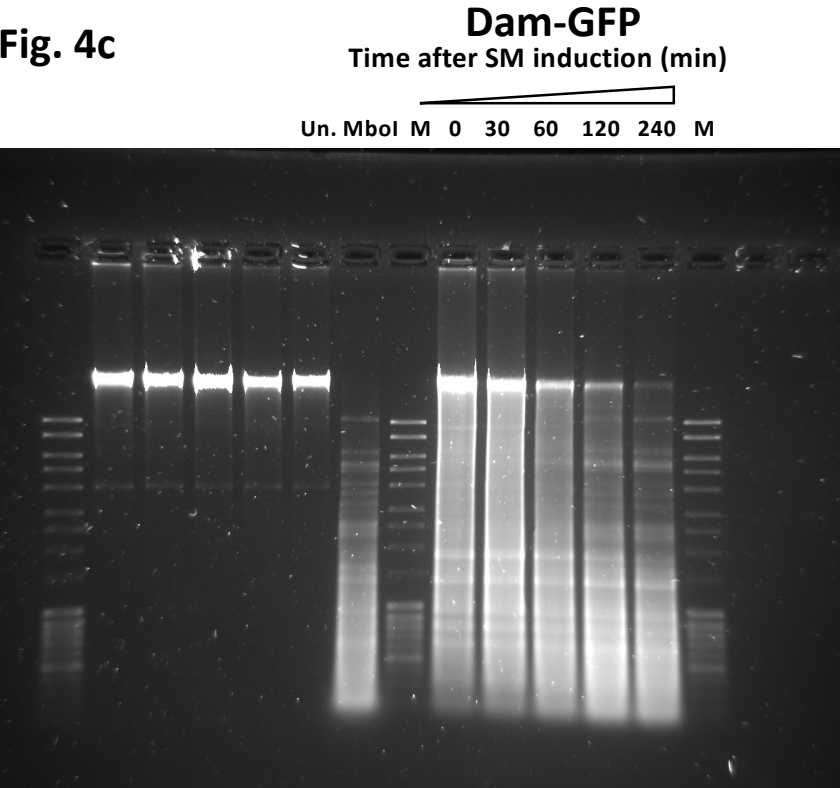

Extended Data Fig. 4d

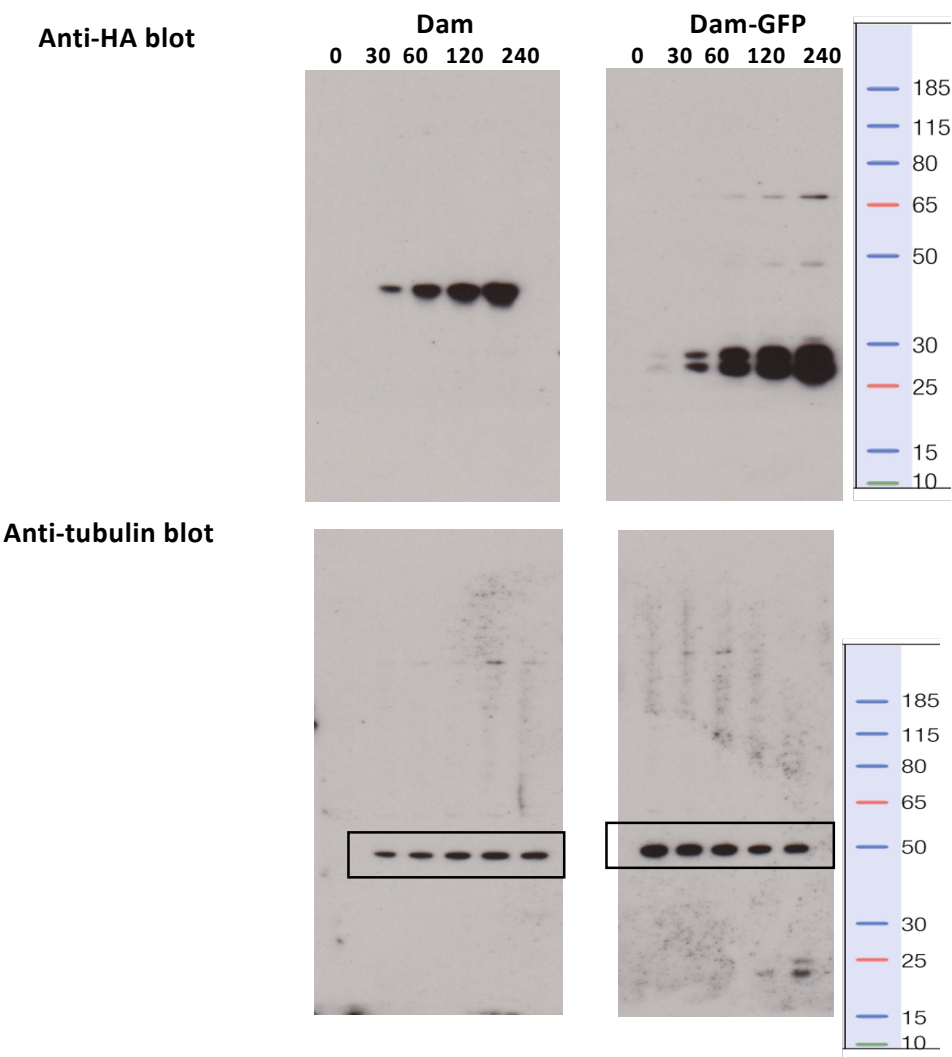

Supplement: Supplementary file 16 — Unprocessed gel and western blots. [file 41594_2024_1318_MOESM16_ESM.pdf]
